# Social Determinants of Health and HIV Diagnosis Rates in U.S. Counties, Comparing Ending the Epidemic (EHE) and Non-EHE Priority Jurisdictions

**DOI:** 10.1007/s10461-025-04865-x

**Published:** 2025-09-08

**Authors:** Elizabeth Lockhart, Elyse Llamocca, Geoff Kahn, Amy Loree, DeAnne Turner

**Affiliations:** 1 Center for Health Policy and Health Services Research, Henry Ford Health + Michigan State University, Detroit, MI USA; 2 College of Nursing, University of South Florida, Tampa, FL USA

**Keywords:** Social determinants of health, HIV, Diagnoses, Ending the epidemic

## Abstract

In the U.S., HIV diagnoses have remained steady over the past decade – despite the availability of condoms and pre-exposure prophylaxis. Factors such as adverse Social Determinants of Health (SDoH) may contribute to the sustained HIV diagnosis rate. This study sought to identify SDoH factors associated with HIV diagnosis rates in U.S. counties and between Ending the Epidemic (EHE) priority jurisdictions and non-EHE jurisdictions. We obtained county-level data from publicly available sources. We fit Poisson regression models to estimate associations between separate county-level SDoH factors and county-level HIV diagnosis rates among 344 U.S. counties and 82 EHE priority jurisdictions. Among all U.S. counties, five factors were associated with HIV diagnosis rates. In all U.S. counties, higher percent of renter-occupied housing with rent at least 30% of household income, percent of population with no health insurance, presence of medically underserved area, and percent of housing units that are overcrowded were associated with HIV diagnosis rates. For three factors (percent of populations with less than a high school education, Index of Dissimilarity, and number of social organizations), associations with HIV diagnosis rates were significantly different between non-EHE and EHE priority jurisdictions. Future research should examine SDoH drivers of HIV diagnoses, including how they impact HIV prevention efforts. Long term, these efforts can help develop novel interventions to reduce HIV transmission.

**Supplementary Information:**

The online version contains supplementary material available at 10.1007/s10461-025-04865-x.

## Introduction

In 2024, there were approximately 1.2 million people with human immunodeficiency virus (HIV) in the United States (U.S.) [1]. Although individual-level biomedical and biobehavioral HIV prevention methods, such as condoms, syringe exchange programs, and Preexposure Prophylaxis (PrEP), are available, HIV diagnoses in the U.S. have remained steady over the past 10–15 years; roughly 35,000 new infections were diagnosed per year from 2018 to 2021 [2]. Previous research has demonstrated that PrEP and other biomedical and biobehavioral interventions can effectively reduce HIV transmission [3–5] yet these alone have not been sufficient in ending the HIV epidemic, suggesting that additional factors may need to be addressed to reduce infections.

Beyond the biomedical factors, social determinants of health (SDoH) may drive HIV diagnoses. SDoH are defined as “conditions in the environments where people are born, live, learn, work, play, worship, and age that affect a wide range of health…outcomes and risks,” [6] that impact health. Examples include education, neighborhood and environment, social and community context, access to quality and affordable health care, and economic status. Research has shown that SDoH can account for 50–80% of the variance in population-level health outcomes [7, 8], while clinical care is estimated to account for 20%; thus, when medical interventions alone are not sufficient to improve health, addressing SDoH may be critical. SDoH are likely key targets for interventions to continue decreasing HIV diagnoses.

There are no known nationwide programs that aim to address social needs, such as food or housing insecurity, specifically for people at increased risk of acquiring HIV. Addressing these adverse SDoH are important, as previous research has found that SDoH such as income inequality are associated with higher HIV diagnosis rates [9]. For example, food insecurity may lead to increased sexual behavior due to increased odds of sex work to obtain money for food [10]. However, these studies are limited, as they were conducted prior to the establishment of Ending the HIV Epidemic (EHE) priority jurisdictions.

Emerging literature extolls the need to address SDoH as a means to improve the HIV care and prevention continuums [11, 12]. However, few studies have empirically examined the relationship between SDoH and HIV diagnoses. Since HIV diagnoses have remained stable over the past decade, we need to examine additional factors that may be driving the sustained level of HIV diagnoses, as well as disparities in HIV diagnoses. The purpose of this study, therefore, is to examine the associations between county-level SDoH factors and HIV diagnosis rates. Additionally, this study seeks to examine the associations between SDoH factors and HIV diagnosis rates in Ending the HIV Epidemic (EHE) priority jurisdictions [13]. It is important to understand if associations between SDoH and HIV diagnosis rates are different in EHE compared to non-EHE jurisdictions and also throughout the U.S. to determine which specific tailored future research and intervention development is needed.

## Methods

### Data Sources

In this cross-sectional study, we obtained county-level variables from publicly available data accessed from the following data sources: the Centers for Disease Control and Prevention National Center for HIV, Viral Hepatitis, STD, and TB Prevention (NCHHSTP) AtlasPlus [14], the Agency for Healthcare Research and Quality (AHRQ) SDoH database [15], the Robert Wood Johnson Foundation County Health Rankings [16], and the Vera Institute of Justice [17]. Additional details regarding how the data was obtained can be accessed through each corresponded reference.

## Measures

### Dependent Variable

The dependent variable of interest was the 2019 county-level HIV diagnosis rate among individuals aged at least 13 years, defined as those diagnoses confirmed by laboratory or clinical evidence within that year [14]. We utilized data from 2019, as this was the most recent data available not directly impacted by the COVID-19 pandemic. To maintain patient confidentiality, rates from counties where small populations and/or numbers of cases make reidentification possible are not publicly available.

### Social Determinants of Health

Our independent variables of interest included county-level factors related to the following SDoH domains, defined based on domains utilized in the AHRQ SDoH database [15]: economic context (*n* = 6 factors), education context (*n* = 1 factor), healthcare context (*n* = 3 factors), physical infrastructure (*n* = 7 factors), and social context (*n* = 1 factor). Table 1 summarizes the specific variables included in each SDoH domain.

**Table 1:** County-Level social determinant of health factors used in analysis

Social determinant of health domain	County-level measure	Source	Time period
Economic Context	Percent aged at least 16 years unemployed	AHRQ SDoH Database	2015–2019
	Gini index, based on household income *0 (perfect income equality) to 1 (perfect income inequality)*	AHRQ SDoH Database	2015–2019
	Percent of households receiving food stamp/Supplemental Nutrition Assistance Program (SNAP) benefits within past 12 months	AHRQ SDoH Database	2015–2019
	Percent of renter-occupied housing units with a rent ≥ 30% of a household’s income	AHRQ SDoH Database	2015–2019
	Percent of median income spent on childcare costs for a household with two children	RWJF County Rankings	2020–2021
	Number of child day care services per 100,000 people	AHRQ SDoH Database	2019
Education Context	Percent aged at least 25 years with less than a high school education	AHRQ SDoH Database	2015–2019
Healthcare Context	Percent of overall population without healthcare insurance	AHRQ SDoH Database	2015–2019
	Number of home healthcare services per 100,000 people	AHRQ SDoH Database	2019
	Presence of Medically Underserved Area within county	AHRQ SDoH Database	2019
Physical Infrastructure	Food Environment Index *0 to 10; higher values indicate a healthier food environment*	RWJF County Rankings	2019
	Percent of housing units lacking complete kitchen or plumbing facilities	RWJF County Rankings	2015–2019
	Percent of housing units that were overcrowded	RWJF County Rankings	2015–2019
	Percent of occupied housing units that were rented	AHRQ SDoH Database	2015–2019
	Percent of housing units with no vehicle available	AHRQ SDoH Database	2015–2019
	Total jail population rate per 100,000 people aged 15 to 64 years	Vera Institute of Justice	2018
	Index of Dissimilarity *0 to 100; higher values indicate more racial/ethnic homogeneity between residents*	AHRQ SDoH Database	2015–2019
Social Context	Number of social associations per 100,000 people	RWJF County Rankings	2019

### Covariates

We adjusted our analyses for county-level demographics (e.g. age and race), HIV-related healthcare access (e.g. PrEP coverage and syringe-exchange program availability), and county population levels. Table S1 lists the complete list of variables included as covariates in our analysis.

### Statistical Analysis

Data was linked across datasets using the Federal Information Processing Standard (FIPS) code for each county. We fit Poisson regression models with robust standard errors, an offset term for the log of the number of individuals aged at least 13 years in each county in 2019 [14], and a random intercept for state to estimate associations between county-level SDoH factors and county-level HIV diagnosis rates. We standardized all continuous independent variables, based on data from all 3,138 counties in the 50 US states and Washington D.C. included in all datasets utilized in our analysis, even if data was missing for that county in at least one dataset. Our final analytic sample was limited to those counties in the United States that had complete data for all SDoH measures, all covariates, and HIV diagnosis rate, which included 344 counties from 37 US states and Washington D.C. The exact number of counties with missing data for each variable is included as a footnote for Table 2. Of the 344 counties with complete data included in our final analytic dataset, 82 counties from 26 US states and Washington D.C. were EHE priority jurisdictions [13]. We compared characteristics between counties included and excluded from the final analytic dataset and between non-EHE and EHE priority jurisdictions included in the final analytic sample using Wilcoxon-Mann-Whitney and chi-squared tests. We fit unadjusted and adjusted models to examine associations between each SDoH factor and county-level HIV diagnosis rate. Due to potential differences in context between non-EHE and EHE priority jurisdictions, we examined differences in the effect of each SDoH factor between non-EHE and EHE priority jurisdictions by fitting models including an interaction term between each SDoH factor and EHE priority jurisdiction status of the county. We accounted for multiple testing through Bonferroni correction, utilizing a significance threshold of α = 0.00048 obtained by dividing α = 0.05 by the total number of statistical tests performed (*n* = 104). All analyses were performed with SAS version 9.4. The Henry Ford Health Institutional Review Board classified this research as exempt from ethical review due to the use of deidentified, publicly available data.

**Table 2:** County-Level characteristics for U.S. Counties included in analysis

	Analytic sample of All United States counties with complete data for all variables (*n* = 344)		Non-EHE priority jurisdiction counties, selected from analytic sample (*n* = 262)	
County-Level Measure	Range/*n* (%)	Mean (SD)	Range/*n* (%)	Mean (SD)
Outcome				
HIV diagnoses per 100,000 people aged at least 13 years	2.03–456.04	30.30 (60.03)	2.03-124.21	13.76 (14.82)
Covariates				
Healthcare Context				
HIV-Related Healthcare Access				
Syringe exchange programs per 100,000 people	0.00–2.95	0.11 (0.27)	0.00-2.95	0.13 (0.29)
Ryan White HIV medical providers per 100,000 people	0.00–2.70	0.33 (0.42)	0.00-2.70	0.27 (0.34)
Substance abuse facilities offering all 3 medication assisted treatment services per 100,000 people	0.00–2.34	0.18 (0.30)	0.00-2.34	0.20 (0.32)
Substance abuse facilities offering HIV testing and accepting Medicaid per 100,000 people	0.00–9.10	0.94 (0.97)	0.00-9.10	0.97 (1.02)
Percent of population with PrEP indicators receiving a PrEP prescription	3.40–91.90	22.78 (12.20)	3.40–63.40	22.67 (11.40)
Medical and diagnostic laboratories per 100,000 people	1.00–22.00	6.15 (2.96)	1.00–22.00	6.07 (3.04)
Social Context				
Demographic Characteristics				
Percent of population female	47.78–53.37	51.04 (0.89)	47.78–53.18	50.94 (0.88)
Percent of population aged 18 to 29 years	10.08–35.85	16.96 (3.66)	10.08–35.85	16.77 (3.86)
Percent of population non-Hispanic Black	0.01–71.23	13.05 (13.09)	0.01–59.30	10.84 (11.44)
Percent of population Hispanic	1.11–95.47	16.28 (15.76)	1.11–95.47	16.41 (16.02)
Percent of population with same-sex unmarried partner	0.01–0.58	0.13 (0.07)	0.01–0.41	0.12 (0.06)
Percent of population with same-sex spouse	0.04–0.64	0.16 (0.07)	0.04–0.43	0.16 (0.06)
County population aged at least 13 years	75,557.00–971,718.00	339,600.33 (215,948.55)	75,557.00-971,718.00	328,863.61 (202,366.36)
County population density (number of people per square mile of land area)	7.59–72,020.87	1,381.91 (4,290.57)	7.59-8,979.38	868.07 (1,080.73)
Rural-Urban Continuum Code				
Counties in metro areas of 1 million population or more	187 (54.36%)		130 (49.6%)	
Counties in metro areas of 250,000 to 1 million population	124 (36.05%)		101 (38.5%)	
Counties in metro areas of fewer than 250,000 population	32 (9.30%)		30 (11.5%)	
Urban population of 20,000 or more, adjacent to a metro area	0 (0.0%)		0 (0.0%)	
Urban population of 20,000 or more, not adjacent to a metro area	1 (0.3%)		1 (0.4%)	
Urban population of 2,500 to 19,999, adjacent to a metro area	0 (0.0%)		0 (0.0%)	
Urban population of 2,500 to 19,999, not adjacent to a metro area	0 (0.0%)		0 (0.0%)	
Completely rural or less than 2,500 urban population, adjacent to a metro area	0 (0.0%)		0 (0.0%)	
Completely rural or less than 2,500 urban population, not adjacent to a metro area	0 (0.0%)		0 (0.0%)	
Social Determinant of Health Factors of Interest				
Economic Context				
Percent of population aged at least 16 years unemployed	2.42–11.87	5.34 (1.50)	2.46–11.87	5.27 (1.52)
Gini Index^b^	0.38–0.60	0.46 (0.03)	0.38–0.54	0.45 (0.03)
Percent of households receiving food stamps/SNAP in past 12 months	1.79–29.07	11.03 (4.78)	1.79–29.07	10.84 (4.91)
Percent of renter-occupied housing units with rent of at least 30% of household income	34.96–64.49	49.61 (4.99)	37.69–63.82	49.67 (4.85)
Percent of median income spent on childcare costs for household with two children	11.16–61.99	25.96 (6.72)	11.16–55.95	26.19 (6.56)
Total number of child day care services per 100,000 people	9.00–51.00	23.85 (8.13)	9.00–51.00	23.56 (8.03)
Education Context				
Percent of population aged at least 25 years with less than high school education	1.87–34.28	10.89 (4.79)	1.87–34.28	10.69 (5.16)
Healthcare Context				
Percent of population with no health insurance coverage	1.83–30.33	8.77 (4.13)	1.83–30.33	8.42 (4.22)
Total number of home healthcare services per 100,000 people	2.00–112.00	10.55 (8.02)	2.00–39.00	9.82 (4.97)
Presence of a Medically Underserved Area				
Yes	46 (13.37%)		41 (15.6%)	
No	298 (86.63%)		221 (84.4%)	
Physical Infrastructure				
Food Environment Index^c^	5.30–10.00	7.96 (0.87)	5.30–10.00	7.98 (0.91)
Percent of housing units lacking complete kitchen or plumbing facilities	0.31–4.29	0.89 (0.38)	0.31–3.85	0.89 (0.35)
Percent of housing units that are overcrowded	0.68–13.80	2.87 (2.09)	0.68–13.80	2.77 (2.08)
Percent of occupied housing units that are rented	18.21–75.93	35.57 (9.25)	18.21–60.97	33.90 (8.35)
Percent of housing units with no vehicle available	2.05–76.99	7.22 (5.86)	2.05–28.90	6.45 (3.11)
Total jail population rate per 100,000 people	60.06–2,004.79	366.70 (221.44)	67.12–2,004.79	375.16 (242.11)
Index of Dissimilarity^d^	14.83–68.84	37.17 (10.43)	14.83–65.04	35.97 (9.71)
Social Context				
Total number of social associations per 100,000 people	18.55–282.25	89.58 (28.87)	18.55–184.69	88.40 (27.02)

	EHE priority jurisdiction counties, selected from analytic sample (*n* = 82) (*n* = 82)		*P*-value comparing Non-EHE and EHE priority jurisdiction counties^a^
County-level measure	Range/*n* (%)	Mean (SD)	
Outcome			
HIV diagnoses per 100,000 people aged at least 13 years	2.97–456.04	83.16 (104.10)	**< 0.0001**
Covariates			
Healthcare Context			
HIV-Related Healthcare Access			
Syringe exchange programs per 100,000 people	0.00–0.79	0.08 (0.16)	0.51
Ryan White HIV medical providers per 100,000 people	0.00–2.66	0.52 (0.57)	**0.0001**
Substance abuse facilities offering all 3 medication assisted treatment services per 100,000 people	0.00–0.92	0.13 (0.17)	0.99
Substance abuse facilities offering HIV testing and accepting Medicaid per 100,000 people	0.00–4.35	0.86 (0.76)	0.63
Percent of population with PrEP indicators receiving a PrEP prescription	5.60–91.90	23.13 (14.53)	0.80
Medical and diagnostic laboratories per 100,000 people	1.00–13.00	6.43 (2.67)	0.20
Social Context			
Demographic Characteristics			
Percent of population female	48.99–53.37	51.38 (0.85)	**< 0.0001**
Percent of population aged 18 to 29 years	12.91–27.90	17.60 (2.87)	0.001
Percent of population non-Hispanic Black	1.06–71.22	20.11 (15.41)	**< 0.0001**
Percent of population Hispanic	1.54–68.49	15.87 (14.97)	0.31
Percent of population with same-sex unmarried partner	0.06–0.58	0.17 (0.09)	**< 0.0001**
Percent of population with same-sex spouse	0.07–0.64	0.18 (0.10)	0.30
County population aged at least 13 years	87,749.00–925,740.00	373,905.44 (252,888.80)	0.45
County population density (number of people per square mile of land area)	107.13–72,020.87	3,023.68 (8,402.86)	**< 0.0001**
Rural-Urban Continuum Code			
Counties in metro areas of 1 million population or more	57 (69.51%)		0.003
Counties in metro areas of 250,000 to 1 million population	23 (28.05%)		
Counties in metro areas of fewer than 250,000 population	2 (2.44%)		
Urban population of 20,000 or more, adjacent to a metro area	0 (0.0%)		
Urban population of 20,000 or more, not adjacent to a metro area	0 (0.0%)		
Urban population of 2,500 to 19,999, adjacent to a metro area	0 (0.0%)		
Urban population of 2,500 to 19,999, not adjacent to a metro area	0 (0.0%)		
Completely rural or less than 2,500 urban population, adjacent to a metro area	0 (0.0%)		
Completely rural or less than 2,500 urban population, not adjacent to a metro area	0 (0.0%)		
Social Determinant of Health Factors of Interest			
Economic Context			
Percent of population aged at least 16 years unemployed	2.42–9.42	5.56 (1.42)	0.05
Gini Index^b^	0.40–0.60	0.48 (0.04)	**< 0.0001**
Percent of households receiving food stamps/SNAP in past 12 months	3.98–25.79	11.66 (4.30)	0.12
Percent of renter-occupied housing units with rent of at least 30% of household income	34.96–64.49	49.42 (5.44)	0.80
Percent of median income spent on childcare costs for household with two children	12.70–61.99	25.21 (7.19)	0.21
Total number of child day care services per 100,000 people	9.00–47.00	24.78 (8.42)	0.34
Education Context			
Percent of population aged at least 25 years with less than high school education	5.21–20.87	11.51 (3.28)	0.002
Healthcare Context			
Percent of population with no health insurance coverage	3.66–21.01	9.87 (3.62)	0.001
Total number of home healthcare services per 100,000 people	2.00–112.00	12.88 (13.62)	0.03
Presence of a Medically Underserved Area			
Yes	5 (6.10%)		0.03
No	77 (93.90%)		
Physical Infrastructure			
Food Environment Index^c^	5.60–9.10	7.89 (0.71)	0.32
Percent of housing units lacking complete kitchen or plumbing facilities	0.32–4.29	0.91 (0.46)	0.69
Percent of housing units that are overcrowded	0.80–11.33	3.20 (2.09)	0.02
Percent of occupied housing units that are rented	19.37–75.93	40.92 (10.01)	**< 0.0001**
Percent of housing units with no vehicle available	2.26–76.99	9.70 (10.29)	0.003
Total jail population rate per 100,000 people	60.06–748.07	339.66 (133.42)	0.96
Index of Dissimilarity^d^	17.76–68.84	41.00 (11.71)	0.001
Social Context			
Total number of social associations per 100,000 people	34.23–282.25	93.35 (34.05)	0.37

^a^Bolded p-values are statistically significant using the Bonferroni corrected significance threshold of α = 0.00048

## Results

### Sample Description

Table S2 describes the characteristics of all counties included in all datasets used in our analyses, while Table 2 describes the characteristics of the counties included in our analytic sample (i.e. all counties with complete data on all variables of interest). Table S3 describes the characteristics of all counties excluded from our analysis and compares them to those included in the analysis. There were significant differences in counties across all SDoH domains. Results are presented as the mean [standard deviation (SD)].

As expected by definition, compared to the 262 counties included in the final analytic sample that were not EHE priority jurisdictions, the 82 counties included in EHE jurisdictions had higher HIV diagnosis rates per 100,000 people aged at least 13 years (non-EHE counties: 13.76 [14.82], EHE counties: 83.16 [104.10]). EHE jurisdictions also had higher rates of Ryan White HIV medical providers per 100,000 people (non-EHE counties: 0.27 [0.34], EHE counties: 0.52 [0.57]). The percent of the population that was female (non-EHE counties: 50.94 [0.88], EHE counties: 51.38 [0.85]), non-Hispanic Black (non-EHE counties: 10.84 [11.44], EHE counties: 20.11 [15.41]), had a same-sex unmarried partner (non-EHE counties: 0.12 [0.06], EHE counties: 0.17 [0.09]) was also higher, as was the population density (non-EHE counties: 868.07 [1,080.73], EHE counties: 3,023.68 [8,402.86]), income inequality (Gini Index: non-EHE counties: 0.45 [0.03], EHE counties: 0.48 [0.04]), and percent of housing units that were rented (non-EHE counties: 33.90 [8.35], EHE counties: 40.92 [10.01]).

## Associations Between County-Level Social Determinants of Health in U.S. Counties and HIV Diagnosis Rates

Table 3 shows the unadjusted and adjusted incidence rate ratio (IRR) and 95% confidence interval (CI) estimates of associations between county-level SDoH factors and HIV diagnosis rates among all 344 counties included in our analysis. An IRR greater than 1.0 indicates a positive association, meaning higher values of the independent variable are linked to higher HIV diagnosis rates. An IRR less than 1.0 indicates a negative association, meaning lower values of the independent variable are linked to higher HIV diagnosis rates. After adjusting for multiple comparisons, 5 SDoH factors across 3 domains (as categorized in Table 1) were significantly associated with county-level HIV diagnosis rate. One variable related to economic context, two variables related to healthcare context, and one variable related to physical infrastructure were positively associated and one variable related to healthcare context was negatively associated with county-level HIV diagnosis rate.

**Table 3:** Associations between County-Level social determinants of health and County-Level HIV diagnosis rate among all united States counties with complete data (*n* = 344)

	IRR (95% CI)^a^		
Social determinant of health factor	Unadjusted	Adjusted^b^	*P*-value from adjusted model^b^
Economic Context			
Percent of population aged at least 16 years unemployed	1.49 (0.99–2.24)	1.13 (0.79–1.60)	0.50
Gini Index^c^	2.01 (1.60–2.54)	1.33 (1.02–1.71)	0.03
Percent of households receiving food stamps/SNAP in past 12 months	1.43 (0.99–2.07)	0.93 (0.71–1.21)	0.59
Percent of renter-occupied housing units with rent of at least 30% of household income	2.37 (1.20–4.71)	1.79 (1.31–2.44)	**0.0003**
Percent of median income spent on childcare costs for household with two children	1.62 (1.14–2.31)	1.10 (0.72–1.66)	0.66
Total number of child day care services per 100,000 people	1.33 (0.60–2.92)	0.83 (0.56–1.25)	0.38
Education Context			
Percent of population aged at least 25 years with less than high school education	1.37 (1.09–1.72)	1.24 (0.88–1.76)	0.22
Healthcare Context			
Ending the HIV Epidemic (EHE) Geographic Focus Area	8.33 (5.05–13.73)	5.08 (2.76–9.35)	**< 0.0001**
Percent of population with no health insurance coverage	1.72 (1.08–2.76)	1.67 (1.38–2.02)	**< 0.0001**
Total number of home healthcare services per 100,000 people	1.75 (0.99–3.09)	1.12 (0.80–1.58)	0.50
Presence of Medically Underserved Area within county	0.26 (0.16–0.41)	0.39 (0.28–0.54)	**< 0.0001**
Physical Infrastructure			
Food Environment Index^d^	1.27 (0.77–2.07)	1.43 (0.98–2.09)	0.07
Percent of housing units lacking complete kitchen or plumbing facilities	1.42 (1.11–1.80)	0.64 (0.36–1.13)	0.12
Percent of housing units that are overcrowded	1.56 (1.09–2.22)	1.33 (1.21–1.47)	**< 0.0001**
Percent of occupied housing units that are rented	1.89 (1.55–2.30)	1.70 (1.14–2.54)	0.01
Percent of housing units with no vehicle available	1.28 (1.14–1.43)	0.97 (0.66–1.45)	0.90
Total jail population rate per 100,000 people	0.24 (0.01–3.94)	0.68 (0.24–1.90)	0.59
Index of Dissimilarity^e^	2.20 (1.54–3.13)	1.30 (1.04–1.61)	0.02
Social Context			
Total number of social associations per 100,000 people	0.70 (0.41–1.20)	1.00 (0.59–1.71)	1.00

^a^All continuous variables were standardized. An IRR greater than 1.0 indicates a positive association, meaning higher values of the independent variable are linked to higher HIV diagnosis rates. An IRR less than 1.0 indicates a negative association, meaning lower values of the independent variable are linked to higher HIV diagnosis rates; ^b^Controlled for demographics (proportion female, aged 18 to 29 years, non-Hispanic Black, Hispanic, with same-sex unmarried partner, with same-sex spouse), access to HIV-related care (syringe exchange programs per 100,000 people, Ryan White HIV medical providers per 100,000 people, substance abuse facilities offering all 3 medication assisted treatment services per 100,000 people, substance abuse facilities offering HIV testing and accepting Medicaid per 100,000 people, preexposure prophylaxis coverage, medical and diagnostic laboratories per 100,000 people), county Rural-Urban Continuum Code, and county population density. Bolded p-values are statistically significant using the Bonferroni corrected significance threshold of α = 0.00048; ^c^Higher Gini index values indicate more income inequality, but are not associated with HIV diagnosis rates; ^d^Higher Food Environment Index values indicate a healthier food environment, but are not associated with HIV diagnosis rates; ^e^Higher Index of Dissimilarity values indicate more racial/ethnic homogeneity between residents, but are not associated with HIV diagnosis rates

### Economic Context

In the full analytic sample, one variable related to economic context was positively associated with HIV diagnosis rate – percent of renter-occupied housing units with rent ≥ 30% of household income (IRR = 1.79 [95% CI: 1.31–2.44]).

### Healthcare Context

In the full analytic sample, two factors related to healthcare context were positively associated with HIV diagnosis rate – being a county identified as an EHE priority jurisdiction (IRR = 5.08 [95% CI: 2.76–9.35]) and percent of population with no health insurance coverage (IRR = 1.67 [95% CI: 1.38–2.02]). One factor was negatively associated with HIV diagnosis rate – presence of a Medically Underserved Area within a county (IRR = 0.39 [95% CI: 0.28–0.54]).

### Physical Infrastructure

In the full analytic sample, one variable related to physical infrastructure was positively associated with increased HIV diagnosis rate – the percent of overcrowded housing units (IRR = 1.33 [95% CI: 1.21–1.47]).

## Associations Between County-Level Social Determinants of Health in EHE and Non-EHE Counties and HIV Diagnosis Rates

Table 4 shows adjusted IRR and 95% confidence interval estimates from the models including an interaction term between each county-level SDoH factor and the county’s EHE priority jurisdiction status. We identified three statistically significant differences between non-EHE and EHE priority jurisdictions after adjusting for multiple comparisons.

**Table 4:** Associations between county-level social determinants of health and county-level HIV diagnosis rate by ending the HIV epidemic (EHE) geographic focus area status among counties with complete data (*n* = 344)

Social determinant of health factor	Non-EHE counties adjusted IRR^a, b^ (95% CI)	EHE counties adjusted IRR^a, b^ (95% CI)	Interaction *P*-value
Economic Context			
Percent of population aged at least 16 years unemployed	1.04 (0.73–1.49)	1.23 (0.83–1.84)	0.57
Gini Index^c^	1.15 (0.93–1.42)	0.91 (0.75–1.11)	0.10
Percent of households receiving food stamps/SNAP in past 12 months	0.95 (0.67–1.35)	1.25 (0.92–1.69)	0.03
Percent of renter-occupied housing units with rent of at least 30% of household income	1.21 (0.80–1.84)	1.85 (1.27–2.70)	0.18
Percent of median income spent on childcare costs for household with two children	1.13 (0.79–1.62)	0.93 (0.66–1.31)	0.32
Total number of child day care services per 100,000 people	1.21 (0.96–1.52)	0.55 (0.33–0.91)	0.01
Education Context			
Percent of population aged at least 25 years with less than high school education	0.91 (0.70–1.19)	1.86 (1.25–2.77)	**< 0.0001**
Healthcare Context			
Percent of population with no health insurance coverage	1.03 (0.79–1.35)	1.64 (1.32–2.03)	0.01
Total number of home healthcare services per 100,000 people	0.92 (0.63–1.36)	1.32 (0.95–1.85)	0.19
Presence of Medically Underserved Area within county	0.75 (0.55–1.01)	0.15 (0.02–0.91)	0.09
Physical Infrastructure			
Food Environment Index^d^	1.19 (0.85–1.67)	1.46 (0.73–2.89)	0.58
Percent of housing units lacking complete kitchen or plumbing facilities	0.44 (0.23–0.83)	0.59 (0.30–1.18)	0.49
Percent of housing units that are overcrowded	0.80 (0.72–0.89)	1.30 (0.96–1.76)	0.01
Percent of occupied housing units that are rented	1.40 (0.95–2.07)	1.71 (1.04–2.80)	0.06
Percent of housing units with no vehicle available	1.13 (0.77–1.66)	0.78 (0.55–1.11)	0.01
Total jail population rate per 100,000 people	1.77 (0.62–5.07)	0.01 (0.00-0.15)	0.001
Index of Dissimilarity^e^	1.42 (1.22–1.65)	0.83 (0.72–0.96)	**< 0.0001**
Social Context			
Total number of social associations per 100,000 people	1.61 (1.12–2.31)	0.24 (0.13–0.42)	**< 0.0001**

^a^All continuous variables were standardized. An IRR greater than 1.0 indicates a positive association, meaning higher values of the independent variable are linked to higher HIV diagnosis rates. An IRR less than 1.0 indicates a negative association, meaning lower values of the independent variable are linked to higher HIV diagnosis rates; ^b^Controlled for demographics (proportion female, aged 18 to 29 years, non-Hispanic Black, Hispanic, with same-sex unmarried partner, with same-sex spouse), access to HIV-related care (syringe exchange programs per 100,000 people, Ryan White HIV medical providers per 100,000 people, substance abuse facilities offering all 3 medication assisted treatment services per 100,000 people, substance abuse facilities offering HIV testing and accepting Medicaid per 100,000 people, preexposure prophylaxis coverage, medical and diagnostic laboratories per 100,000 people), county Rural-Urban Continuum Code, and county population density. Bolded p-values are statistically significant using the Bonferroni corrected significance threshold of α = 0.00048; ^c^Higher Gini index values indicate more income inequality, but are not associated with HIV diagnosis rates; ^d^Higher Food Environment Index values indicate a healthier food environment, but are not associated with HIV diagnosis rates; ^e^Higher Index of Dissimilarity values indicate more racial/ethnic homogeneity between residents, and are associated with higher HIV diagnosis rates in non-EHE jurisdictions and lower HIV diagnosis rates in EHE jurisdictions

### Education Context

The effect on HIV diagnosis rates of the single variable included in our analysis related to education context differed between non-EHE and EHE priority jurisdictions. In non-EHE priority jurisdictions, the percent of the population aged ≥ 25 years with less than a high school education was not associated with HIV diagnosis rate (IRR = 0.91 [95% CI: 0.70–1.19]), but the same variable was positively associated with HIV diagnosis rate (IRR = 1.86 [95% CI: 1.25–2.77]) in EHE priority jurisdictions.

### Physical Infrastructure

The effect of residential segregation on HIV diagnosis rate differed between non-EHE and EHE priority jurisdictions. In non-EHE priority jurisdictions, increased residential segregation was positively associated with county-level HIV diagnosis rate (IRR = 1.42 [95% CI: 1.22–1.65]), while increased residential segregation was negatively associated with county-level HIV diagnosis rate in EHE priority jurisdictions (IRR = 0.83 [95% CI: 0.72–0.96]).

### Social Context

The effect of the total number of social organizations per 100,000 people, differed by county EHE priority jurisdiction status. In non-EHE priority jurisdictions, a higher social organization rate was positively associated with HIV diagnosis rate (IRR = 1.61 [95% CI: 1.12–2.31]), but it was negatively associated with HIV diagnosis rate in EHE priority jurisdictions s (IRR = 0.24 [95% CI: 0.13–0.42]).

## Discussion

This exploratory study used county-level data to determine associations between SDoH and HIV diagnosis rates both among US counties and between non-EHE and EHE priority jurisdictions. After adjusting for (1) county-level demographics such as age and race; (2) HIV-related healthcare access, such as PrEP coverage and syringe exchange program availability; and (3) population levels, such as population density, we found five SDoH factors associated with HIV diagnosis rates among all U.S. counties with available data; the associations with HIV diagnosis rates were significantly different between non-EHE and EHE priority jurisdictions for three SDoH factors. This is one of the first studies to empirically examine how multiple SDoH impact HIV diagnosis rates at the county-level and between non-EHE and EHE priority jurisdictions specifically, and thus may identify future areas for intervention.

A novel aspect of our analysis was our examination of associations between SDoH and HIV diagnosis rates in non-EHE and EHE priority jurisdictions. In 2019, the U.S. government identified 57 counties and states where more than 50% of HIV diagnoses occurred to focus prevention efforts for maximum impact. Identifying the SDoH factors associated with HIV diagnosis rates in both EHE and non-EHE jurisdictions is important for developing potential targeted intervention efforts. Importantly, our study was not designed to understand causal associations, or *why* certain SDoH factors were associated with higher HIV diagnosis rates; future research should attempt to do so.

We found that more social organizations were associated with lower HIV diagnosis rates in EHE counties compared to non-EHE priority jurisdictions. While there is a breadth of research that has investigated social connections and social isolation, and how they relate to HIV-related outcomes (CD4 counts, engagement in treatment) [18–20], no known research has investigated the relationship between the number of social organizations, the support they may provide and HIV diagnoses. Social organizations may provide numerous types of support to individuals related to HIV diagnoses, including social cohesion, social capital, social support. Lack of social connection has been associated with mental health issues such as loneliness and depression [21–23] and may also be important for reducing HIV diagnoses as well. Future research should examine how social connectedness and the role of social organizations impacts HIV diagnoses and why the association differs in non-EHE priority jurisdictions.

Lack of education has been found to be associated with increased HIV rates in other studies – both at the individual level [24] and county levels throughout the U.S [25]., but has not been associated specifically with EHE jurisdictions. At the county-level, education may be a proxy for other factors, such as lack of investment and divestment in communities. Lower educational attainment often leads to less pay and fewer employment opportunities. While related factors were not significantly associated with HIV diagnoses, educational attainment may be the underlying driver. Increasing investment in education and alternative education programs to help people receive their high school diploma may also provide additional protective factors for HIV acquisition. Further research should examine if other factors not captured in our analyses contribute to increased vulnerability among people without a high school diploma.

Nationally, factors associated with housing (i.e., affordability and overcrowding), health insurance coverage, and health care resources were also associated with HIV diagnoses rates. There are currently some national programs available for people with HIV (PWH) to address adverse SDoH, such as the Housing Opportunities for People with AIDS (HOPWA) program and Ryan White Program for comprehensive medical care. These types of broad, nationwide, programs are important, as PWH on Ryan White are more likely to be virally suppressed than PWH not receiving Ryan White assistance [26, 27]. Fewer programs exist to address adverse SDoH in people who are disproportionately at risk of acquiring HIV. Patient assistance programs for PrEP assist individuals who either do not have health insurance or cannot otherwise afford PrEP medication. Additionally, the Affordable Care Act covers preventive services, such as PrEP; however, the extent to which all related costs are covered in practice is debated [28].

Our work points to the importance of understanding how SDoH are impacting current efforts to reduce HIV diagnosis rates. Inherent in HIV diagnosis rates are factors associated with both transmission and clinical diagnostic testing. Research has found that HIV-transmission risk varies dependent on the exposure [29]. Additionally, estimates indicate that 13% of people with HIV do not know their status [30], either because they have not been tested or do not have access to testing. It is important to understand differences in HIV diagnoses and incidence and how SDoH impact each. While there are calls for focusing on SDoH in preventing HIV [11], future research should elucidate which specific SDoH variables are most likely to be causally associated with HIV diagnoses, as correlations between SDoH variables may confound their associations with HIV, and then explore the mechanisms of *how* SDoH impact HIV prevention. For example, qualitative research could elucidate how lack of social organizations leads to increased HIV risk. Additionally, longitudinal, individual-level data should be combined with county-level data for a more nuanced understanding of associations between SDoH and HIV risk.

In combination with previous research examining associations between county and state-level SDoH and HIV diagnoses and incidence rates [9, 31], our findings suggest the importance of understanding which of these SDoH are most likely to lead to increased HIV transmission risk and conversely which factors may be protective and reduce HIV transmission risk. It is imperative to understand the directionality as well – whether adverse SDoH lead to increased HIV incidence or whether areas with increased HIV incidence lead to individuals having adverse SDoH. Previous research has found associations between many different SDoH and outcomes of people living with HIV [32–34], however there is a dearth of research that examines the directionality between SDoH and HIV diagnoses and incidence rates. For example, one study found that people with HIV who experienced 4 or more SDoH indicators were 3.6 times as likely to miss a medical appointment in the previous year and 20% less likely to achieve durable viral suppression in the previous year [34]. Additionally, our work points to the importance of understanding how SDoH are impacting current efforts to reduce HIV transmission. While there are calls for focusing on SDoH in preventing HIV [11], future research should explore the mechanisms of *how* SDoH impact HIV prevention. For example, qualitative research could elucidate how economic or housing insecurity lead to increased HIV risk. While some studies have examined how adverse SDoH impact PrEP interest among youth [35, 36], they have not examined HIV risk generally nor in adult populations. Once we better understand which factors are driving risk and how they impact HIV diagnoses and incidence rates, interventions should be developed to address those SDoH factors.

This study has limitations. First, the publicly available data utilized in this analysis had several constraints. We utilized data that was several years old, however we chose this data to make use of the most recent data available that was not impacted by the COVID-19 pandemic. Several of the SDoH measures included our analysis had a different reference population (e.g., the total jail population rate was per 100,000 individuals aged 15 to 64 years) than our outcome (HIV diagnoses per 100,000 people aged at least 13 years), which may limit the direct impact of the exposure measured on the full population captured in the outcome. Although we utilized multiple national datasets of county-level measures of diverse SDoH in our analysis, we were unable to account for individual experiences with adverse SDoH. To achieve a more robust understanding of how HIV diagnosis rates are related to SDoH, future research should capture individual-level SDoH and ultimately combine that with county-level SDoH data. Using a conceptual or theoretical framework, such as the Syndemic Framework [37], may lead to further insights. As leading healthcare quality and regulatory agencies begin to require SDoH screening, documentation, and intervention for patients [38–42], there will be ample opportunity to combine data that healthcare systems collect with publicly available data to understand the unique and combined contributions of individual and community level drivers of HIV transmission and diagnosis. Second, this study was exploratory and did not determine causality or temporal directionality between SDoH and HIV diagnosis rates. Future research should examine causal associations and directionality between SDoH factors and HIV incidence, particularly in EHE priority jurisdictions. Qualitative methods should be utilized in future research to help understand the mechanisms linking SDoH and HIV incidence. For example, qualitative methods may help elucidate potential participant preferences for developing interventions that will address adverse SDoH for people at increased risk of acquiring HIV. Third, limited sample size or variability within our sample after stratifying for EHE priority jurisdiction status may have limited our power to detect statistically significant associations. Finally, although we examined relationships between numerous county-level SDoH variables and HIV diagnosis rates in this analysis, it is likely that certain variables are correlated or may be proxies for other factors driving HIV incidence. Elucidation of such relationships is necessary in future research exploring causal associations between SDoH and HIV incidence.

As HIV prevention efforts diversify to include methods beyond biomedical and biobehavioral prevention, it will be important to understand the SDoH factors associated with HIV transmission at the county-level and among EHE priority jurisdictions. This study sheds an initial light on the SDoH factors that are positively and negatively associated with HIV diagnosis rates in the U.S.

## Supplementary Information

Below is the link to the electronic supplementary material.

Supplementary Material 1
