# Supplementary material for: Social Determinants of Health and HIV Diagnosis Rates in U.S. Counties, Comparing Ending the Epidemic (EHE) and Non-EHE Priority Jurisdictions
Source: AIDS Behav. 2025 Sep 8;30(1):270–81. doi: 10.1007/s10461-025-04865-x (PMC12509699; doi:10.1007/s10461-025-04865-x)
Supplement: Supplementary file 1 — Supplementary Material 1 [file 10461_2025_4865_MOESM1_ESM.docx]

Table S1. County-Level Covariates Used in Analysis

| **Domain** | **County-Level Measure** | **Source** | **Time Period** |
| --- | --- | --- | --- |
| *Healthcare Context* | | | |
| HIV-Related Healthcare Access | Syringe exchange programs per 100,000 people | AHRQ SDoH Database | 2019 |
|  | Ryan White HIV medical providers per 100,000 people | AHRQ SDoH Database | 2019 |
|  | Substance abuse facilities offering all 3 medication assisted treatment services per 100,000 people  (buprenorphine, methadone, naltrexone) | AHRQ SDoH Database | 2019 |
|  | Substance abuse facilities offering HIV testing and accepting Medicaid per 100,000 people | AHRQ SDoH Database | 2019 |
|  | Percent of population with PrEP indicators aged at least 16 years receiving a PrEP prescription | NCHHSTP AtlasPlus | 2019 |
|  | Medical and diagnostic laboratories per 100,000 people | AHRQ SDoH Database | 2019 |
| *Social Context* | | | |
| Demographic Characteristics | Percent of population female | AHRQ SDoH Database | 2015-2019 |
|  | Percent of population aged 18 to 29 years | AHRQ SDoH Database | 2015-2019 |
|  | Percent of population non-Hispanic Black | AHRQ SDoH Database | 2015-2019 |
|  | Percent of population Hispanic | AHRQ SDoH Database | 2015-2019 |
|  | Percent of population with same-sex unmarried partner | AHRQ SDoH Database | 2015-2019 |
|  | Percent of population with same-sex spouse | AHRQ SDoH Database | 2015-2019 |
|  | County population aged at least 13 years (utilized as an offset term in Poisson regression model) | NCHHSTP AtlasPlus | 2019 |
|  | County population density (number of people per square mile of land area) | AHRQ SDoH Database | *Population Number:* 2015-2019  *Area:* Read definition in Data Dictionary |
|  | Rural-Urban Continuum Code | AHRQ SDoH Database | 2013 |

Table S2. County-Level Characteristics for All U.S. Counties

|  | **All United States Counties Included in All Utilized Datasets^a^ (n=3,138)** | |
| --- | --- | --- |
| **County-Level Measure** | **Range / n (%)** | **Mean (SD)** |
| *Outcome* |  |  |
| HIV diagnoses per 100,000 people aged at least 13 years | 0.00-456.04 | 8.32 (28.09) |
| *Covariates* |  |  |
| *Healthcare Context* |  |  |
| *HIV-Related Healthcare Access* |  |  |
| Syringe exchange programs per 100,000 people | 0.00-20.26 | 0.11 (0.72) |
| Ryan White HIV medical providers per 100,000 people | 0.00-22.71 | 0.19 (1.04) |
| Substance abuse facilities offering all 3 medication assisted treatment services per 100,000 people | 0.00-31.51 | 0.09 (0.68) |
| Substance abuse facilities offering HIV testing and accepting Medicaid per 100,000 people | 0.00-59.93 | 0.89 (2.65) |
| Percent of population with PrEP indicators receiving a PrEP prescription | 3.40-91.90 | 23.15 (12.45) |
| Medical and diagnostic laboratories per 100,000 people | 1.00-54.00 | 6.70 (4.30) |
| *Social Context* |  |  |
| *Demographic Characteristics* |  |  |
| Percent of population female | 27.28-57.19 | 49.91 (2.35) |
| Percent of population aged 18 to 29 years | 4.33-52.64 | 14.71 (4.13) |
| Percent of population non-Hispanic Black | 0.00-87.15 | 8.31 (13.89) |
| Percent of population Hispanic | 0.00-99.17 | 9.39 (13.84) |
| Percent of population with same-sex unmarried partner | 0.00-0.85 | 0.07 (0.08) |
| Percent of population with same-sex spouse | 0.00-1.16 | 0.12 (0.10) |
| County population aged at least 13 years | 87.00-971,718.00 | 67,434.88 (129,004.96) |
| County population density (number of people per square mile of land area) | 0.04-72,020.87 | 239.82 (1,514.26) |
| Rural-Urban Continuum Code |  |  |
| Counties in metro areas of 1 million population or more | 428 (13.64%) |  |
| Counties in metro areas of 250,000 to 1 million population | 378 (12.05%) |  |
| Counties in metro areas of fewer than 250,000 population | 356 (11.34%) |  |
| Urban population of 20,000 or more, adjacent to a metro area | 214 (6.82%) |  |
| Urban population of 20,000 or more, not adjacent to a metro area | 92 (2.93%) |  |
| Urban population of 2,500 to 19,999, adjacent to a metro area | 593 (18.90%) |  |
| Urban population of 2,500 to 19,999, not adjacent to a metro area | 433 (13.80%) |  |
| Completely rural or less than 2,500 urban population, adjacent to a metro area | 220 (7.01%) |  |
| Completely rural or less than 2,500 urban population, not adjacent to a metro area | 424 (13.51%) |  |
| *Social Determinant of Health Factors of Interest* |  |  |
| *Economic Context* |  |  |
| Percent of population aged at least 16 years unemployed | 0.00-27.15 | 5.29 (2.65) |
| Gini Index^b^ | 0.30-0.71 | 0.45 (0.04) |
| Percent of households receiving food stamps/SNAP in past 12 months | 0.00-59.22 | 12.76 (6.40) |
| Percent of renter-occupied housing units with rent of at least 30% of household income | 0.00-88.10 | 43.53 (9.61) |
| Percent of median income spent on childcare costs for household with two children | 10.15-61.99 | 25.31 (6.33) |
| Total number of child day care services per 100,000 people | 4.00-254.00 | 26.07 (16.34) |
| *Education Context* |  |  |
| Percent of population aged at least 25 years with less than high school education | 1.12-73.56 | 13.04 (6.26) |
| *Healthcare Context* |  |  |
| Percent of population with no health insurance coverage | 0.00-46.30 | 9.64 (5.11) |
| Total number of home healthcare services per 100,000 people | 2.00-166.00 | 13.20 (12.46) |
| Presence of a Medically Underserved Area |  |  |
| Yes | 1,543 (49.17%) |  |
| No | 1,595 (50.83%) |  |
| *Physical Infrastructure* |  |  |
| Food Environment Index^c^ | 0.00-10.00 | 7.44 (1.15) |
| Percent of housing units lacking complete kitchen or plumbing facilities | 0.00-36.81 | 1.23 (1.63) |
| Percent of housing units that are overcrowded | 0.00-46.96 | 2.41 (2.37) |
| Percent of occupied housing units that are rented | 6.94-100.00 | 28.37 (8.25) |
| Percent of housing units with no vehicle available | 0.00-87.99 | 6.24 (4.26) |
| Total jail population rate per 100,000 people | 0.00-26,654.55 | 610.73 (1,368.72) |
| Index of Dissimilarity^d^ | 0.15-91.63 | 30.89 (13.10) |
| *Social Context* |  |  |
| Total number of social associations per 100,000 people | 0.00-554.02 | 114.63 (59.04) |

^a^Missing: HIV incidence rate: n=1,072; Population aged at least 13 years: n=1; PrEP coverage: n=2,758; Medical and diagnostic labs: n=2,377; Food Environment Index: n=33; Jail population: n=177; Dissimilarity Index: n=341; Childcare cost burden: n=2; Child day care centers: n=969, Home health care services: n=1,731; ^b^Gini Index ranges from 0 (best) to 1 (worst); ^c^Food Environment Index ranges from 0 (least healthy) to 10 (most healthy); ^d^Index of Dissimilarity ranges from 0 (best) to 100 (worst)

Table S3. Comparison of Counties Included and Not Included in Final Analysis Sample

|  | **Counties Not Included in Final Analytic Sample (n=2,794)** | | **P-value Comparing Counties Included and Excluded from Analytic Sample^a^** |
| --- | --- | --- | --- |
| **County-Level Measure** | **Range / n (%)** | **Mean (SD)** |  |
| *Outcome* |  |  |  |
| HIV diagnoses per 100,000 people aged at least 13 years | 0.00-168.03 | 3.93 (10.62) | **<0.0001** |
| *Covariates* |  |  |  |
| *Healthcare Context* |  |  |  |
| *HIV-Related Healthcare Access* |  |  |  |
| Syringe exchange programs per 100,000 people | 0.00-20.26 | 0.11 (0.75) | **<0.0001** |
| Ryan White HIV medical providers per 100,000 people | 0.00-22.71 | 0.17 (1.09) | **<0.0001** |
| Substance abuse facilities offering all 3 medication assisted treatment services per 100,000 people | 0.00-31.51 | 0.08 (0.71) | **<0.0001** |
| Substance abuse facilities offering HIV testing and accepting Medicaid per 100,000 people | 0.00-59.93 | 0.88 (2.79) | **<0.0001** |
| Percent of population with PrEP indicators receiving a PrEP prescription | 8.10-62.10 | 26.70 (14.34) | 0.14 |
| Medical and diagnostic laboratories per 100,000 people | 1.00-54.00 | 7.15 (5.11) | 0.03 |
| *Social Context* |  |  |  |
| *Demographic Characteristics* |  |  |  |
| Percent of population female | 27.28-57.19 | 49.77 (2.44) | **<0.0001** |
| Percent of population aged 18 to 29 years | 4.33-52.64 | 14.43 (4.10) | **<0.0001** |
| Percent of population non-Hispanic Black | 0.00-87.15 | 7.73 (13.87) | **<0.0001** |
| Percent of population Hispanic | 0.00-99.17 | 8.54 (13.35) | **<0.0001** |
| Percent of population with same-sex unmarried partner | 0.00-0.85 | 0.07 (0.08) | **<0.0001** |
| Percent of population with same-sex spouse | 0.00-1.16 | 0.11 (0.11) | **<0.0001** |
| County population aged at least 13 years | 87.00-820,521.00 | 33,913.61 (52,087.22) | **<0.0001** |
| County population density (number of people per square mile of land area) | 0.04-10,556.80 | 99.20 (366.37) | **<0.0001** |
| Rural-Urban Continuum Code |  |  |  |
| Counties in metro areas of 1 million population or more | 241 (8.63%) |  | **<0.0001** |
| Counties in metro areas of 250,000 to 1 million population | 254 (9.09%) |  |  |
| Counties in metro areas of fewer than 250,000 population | 324 (11.60%) |  |  |
| Urban population of 20,000 or more, adjacent to a metro area | 214 (7.66%) |  |  |
| Urban population of 20,000 or more, not adjacent to a metro area | 91 (3.26%) |  |  |
| Urban population of 2,500 to 19,999, adjacent to a metro area | 593 (21.22%) |  |  |
| Urban population of 2,500 to 19,999, not adjacent to a metro area | 433 (15.50%) |  |  |
| Completely rural or less than 2,500 urban population, adjacent to a metro area | 220 (7.87%) |  |  |
| Completely rural or less than 2,500 urban population, not adjacent to a metro area | 424 (15.18%) |  |  |
| *Social Determinant of Health Factors of Interest* |  |  |  |
| *Economic Context* |  |  |  |
| Percent of population aged at least 16 years unemployed | 0.00-27.15 | 5.28 (2.76) | 0.01 |
| Gini Index^b^ | 0.30-0.71 | 0.44 (0.04) | **<0.0001** |
| Percent of households receiving food stamps/SNAP in past 12 months | 0.00-59.22 | 12.97 (6.54) | **<0.0001** |
| Percent of renter-occupied housing units with rent of at least 30% of household income | 0.00-88.10 | 42.78 (9.78) | **<0.0001** |
| Percent of median income spent on childcare costs for household with two children | 10.15-60.69 | 25.23 (6.28) | 0.06 |
| Total number of child day care services per 100,000 people | 4.00-254.00 | 26.49 (17.43) | 0.74 |
| *Education Context* |  |  |  |
| Percent of population aged at least 25 years with less than high school education | 1.12-73.56 | 13.31 (6.37) | **<0.0001** |
| *Healthcare Context* |  |  |  |
| Percent of population with no health insurance coverage | 0.00-46.30 | 9.75 (5.21) | 0.01 |
| Total number of home healthcare services per 100,000 people | 2.00-166.00 | 14.06 (13.48) | **<0.0001** |
| Presence of a Medically Underserved Area |  |  |  |
| Yes | 1,497 (53.58%) |  | **<0.0001** |
| No | 1,297 (46.42%) |  |  |
| *Physical Infrastructure* |  |  |  |
| Food Environment Index^c^ | 0.00-10.00 | 7.38 (1.16) | **<0.0001** |
| Percent of housing units lacking complete kitchen or plumbing facilities | 0.00-36.81 | 1.27 (1.72) | **<0.0001** |
| Percent of housing units that are overcrowded | 0.00-46.96 | 2.35 (2.39) | **<0.0001** |
| Percent of occupied housing units that are rented | 6.94-100.00 | 27.49 (7.67) | **<0.0001** |
| Percent of housing units with no vehicle available | 0.00-87.99 | 6.12 (4.00) | **<0.0001** |
| Total jail population rate per 100,000 people | 0.00-26,654.55 | 642.80 (1,450.67) | **<0.0001** |
| Index of Dissimilarity^d^ | 0.15-91.63 | 30.01 (13.19) | **<0.0001** |
| *Social Context* |  |  |  |
| Total number of social associations per 100,000 people | 0.00-554.02 | 117.71 (61.04) | **<0.0001** |

^a^Bolded p-values are statistically significant using the Bonferroni corrected significance threshold of α=0.00048; ^b^Gini Index ranges from 0 (best) to 1 (worst); ^c^Food Environment Index ranges from 0 (least healthy) to 10 (most healthy); ^d^Index of Dissimilarity ranges from 0 (best) to 100 (worst)

Table S4. Analytic Sample Description

| Geographic Area | # of Counties:  Total Analytic Sample | # of Counties:  Non-EHE Priority Jurisdiction Areas | # of Counties:  EHE Priority Jurisdiction Areas |
| --- | --- | --- | --- |
| Alabama | 6 | 0 | 6 |
| Arizona | 5 | 4 | 1 |
| Arkansas | 3 | 0 | 3 |
| California | 30 | 22 | 8 |
| Colorado | 7 | 7 | 0 |
| Florida | 31 | 24 | 7 |
| Georgia | 19 | 15 | 4 |
| Illinois | 4 | 3 | 1 |
| Indiana | 10 | 9 | 1 |
| Iowa | 3 | 3 | 0 |
| Kansas | 3 | 3 | 0 |
| Kentucky | 3 | 0 | 3 |
| Louisiana | 8 | 6 | 2 |
| Maryland | 9 | 6 | 3 |
| Massachusetts | 5 | 4 | 1 |
| Michigan | 8 | 7 | 1 |
| Minnesota | 5 | 5 | 0 |
| Mississippi | 5 | 0 | 5 |
| Missouri | 7 | 0 | 7 |
| Nebraska | 2 | 2 | 0 |
| Nevada | 2 | 1 | 1 |
| New Jersey | 16 | 14 | 2 |
| New Mexico | 4 | 4 | 0 |
| New York | 14 | 13 | 1 |
| North Carolina | 18 | 17 | 1 |
| Ohio | 14 | 11 | 3 |
| Oklahoma | 3 | 0 | 3 |
| Oregon | 5 | 5 | 0 |
| Pennsylvania | 17 | 16 | 1 |
| South Carolina | 9 | 0 | 9 |
| Tennessee | 9 | 8 | 1 |
| Texas | 28 | 23 | 5 |
| Utah | 3 | 3 | 0 |
| Virginia | 13 | 13 | 0 |
| Washington | 8 | 7 | 1 |
| Washington D.C. | 1 | 0 | 1 |
| West Virginia | 1 | 1 | 0 |
| Wisconsin | 6 | 6 | 0 |
