# Supplementary material for: Social Determinants of Health and HIV Diagnosis Rates in U.S. Counties, Comparing Ending the Epidemic (EHE) and Non-EHE Priority Jurisdictions
Source: AIDS Behav. Author manuscript; Available in PMC 2025 Oct 10. (PMC12509699; doi:10.1007/s10461-025-04865-x)
Supplement: Supplementary file 2 [file NIHMS2110400-supplement-Supplemental_Table_2.docx]

Table S2. County-Level Characteristics for All U.S. Counties

|  | **All United States Counties Included in All Utilized Datasets**^**a**^ **(n=3,138)** |  |
| --- | --- | --- |
| **County-Level Measure** | **Range / n (%)** | **Mean (SD)** |
| *Outcome* |  |  |
| HIV diagnoses per 100,000 people aged at least 13 years | 0.00-456.04 | 8.32 (28.09) |
| *Covariates* |  |  |
| *Healthcare Context* |  |  |
| *HIV-Related Healthcare Access* |  |  |
| Syringe exchange programs per 100,000 people | 0.00-20.26 | 0.11 (0.72) |
| Ryan White HIV medical providers per 100,000 people | 0.00-22.71 | 0.19 (1.04) |
| Substance abuse facilities offering all 3 medication assisted treatment services per 100,000 people | 0.00-31.51 | 0.09 (0.68) |
| Substance abuse facilities offering HIV testing and accepting Medicaid per 100,000 people | 0.00-59.93 | 0.89 (2.65) |
| Percent of population with PrEP indicators receiving a PrEP prescription | 3.40-91.90 | 23.15 (12.45) |
| Medical and diagnostic laboratories per 100,000 people | 1.00-54.00 | 6.70 (4.30) |
| *Social Context* |  |  |
| *Demographic Characteristics* |  |  |
| Percent of population female | 27.28-57.19 | 49.91 (2.35) |
| Percent of population aged 18 to 29 years | 4.33-52.64 | 14.71 (4.13) |
| Percent of population non-Hispanic Black | 0.00-87.15 | 8.31 (13.89) |
| Percent of population Hispanic | 0.00-99.17 | 9.39 (13.84) |
| Percent of population with same-sex unmarried partner | 0.00-0.85 | 0.07 (0.08) |
| Percent of population with same-sex spouse | 0.00-1.16 | 0.12 (0.10) |
| County population aged at least 13 years | 87.00-971,718.00 | 67,434.88 (129,004.96) |
| County population density (number of people per square mile of land area) | 0.04-72,020.87 | 239.82 (1,514.26) |
| Rural-Urban Continuum Code |  |  |
| Counties in metro areas of 1 million population or more | 428 (13.64%) |  |
| Counties in metro areas of 250,000 to 1 million population | 378 (12.05%) |  |
| Counties in metro areas of fewer than 250,000 population | 356 (11.34%) |  |
| Urban population of 20,000 or more, adjacent to a metro area | 214 (6.82%) |  |
| Urban population of 20,000 or more, not adjacent to a metro area | 92 (2.93%) |  |
| Urban population of 2,500 to 19,999, adjacent to a metro area | 593 (18.90%) |  |
| Urban population of 2,500 to 19,999, not adjacent to a metro area | 433 (13.80%) |  |
| Completely rural or less than 2,500 urban population, adjacent to a metro area | 220 (7.01%) |  |
| Completely rural or less than 2,500 urban population, not adjacent to a metro area | 424 (13.51%) |  |
| *Social Determinant of Health Factors of Interest* |  |  |
| *Economic Context* |  |  |
| Percent of population aged at least 16 years unemployed | 0.00-27.15 | 5.29 (2.65) |
| Gini Index^b^ | 0.30-0.71 | 0.45 (0.04) |
| Percent of households receiving food stamps/SNAP in past 12 months | 0.00-59.22 | 12.76 (6.40) |
| Percent of renter-occupied housing units with rent of at least 30% of household income | 0.00-88.10 | 43.53 (9.61) |
| Percent of median income spent on childcare costs for household with two children | 10.15-61.99 | 25.31 (6.33) |
| Total number of child day care services per 100,000 people | 4.00-254.00 | 26.07 (16.34) |
| *Education Context* |  |  |
| Percent of population aged at least 25 years with less than high school education | 1.12-73.56 | 13.04 (6.26) |
| *Healthcare Context* |  |  |
| Percent of population with no health insurance coverage | 0.00-46.30 | 9.64 (5.11) |
| Total number of home healthcare services per 100,000 people | 2.00-166.00 | 13.20 (12.46) |
| Presence of a Medically Underserved Area |  |  |
| Yes | 1,543 (49.17%) |  |
| No | 1,595 (50.83%) |  |
| *Physical Infrastructure* |  |  |
| Food Environment Index^c^ | 0.00-10.00 | 7.44 (1.15) |
| Percent of housing units lacking complete kitchen or plumbing facilities | 0.00-36.81 | 1.23 (1.63) |
| Percent of housing units that are overcrowded | 0.00-46.96 | 2.41 (2.37) |
| Percent of occupied housing units that are rented | 6.94-100.00 | 28.37 (8.25) |
| Percent of housing units with no vehicle available | 0.00-87.99 | 6.24 (4.26) |
| Total jail population rate per 100,000 people | 0.00-26,654.55 | 610.73 (1,368.72) |
| Index of Dissimilarity^d^ | 0.15-91.63 | 30.89 (13.10) |
| *Social Context* |  |  |
| Total number of social associations per 100,000 people | 0.00-554.02 | 114.63 (59.04) |

^a^Missing: HIV incidence rate: n=1,072; Population aged at least 13 years: n=1; PrEP coverage: n=2,758; Medical and diagnostic labs: n=2,377; Food Environment Index: n=33; Jail population: n=177; Dissimilarity Index: n=341; Childcare cost burden: n=2; Child day care centers: n=969, Home health care services: n=1,731; ^b^Gini Index ranges from 0 (best) to 1 (worst); ^c^Food Environment Index ranges from 0 (least healthy) to 10 (most healthy); ^d^Index of Dissimilarity ranges from 0 (best) to 100 (worst)
