# Supplementary material for: Social Determinants of Health and HIV Diagnosis Rates in U.S. Counties, Comparing Ending the Epidemic (EHE) and Non-EHE Priority Jurisdictions
Source: AIDS Behav. Author manuscript; Available in PMC 2025 Oct 10. (PMC12509699; doi:10.1007/s10461-025-04865-x)
Supplement: Supplementary file 3 [file NIHMS2110400-supplement-Supplemental_Table_3.docx]

Table S3. Comparison of Counties Included and Not Included in Final Analysis Sample

|  | **Counties Not Included in Final Analytic Sample (n=2,794)** |  | **P-value Comparing Counties Included and Excluded from Analytic Sample**^**a**^ |
| --- | --- | --- | --- |
| **County-Level Measure** | **Range / n (%)** | **Mean (SD)** |  |
| *Outcome* |  |  |  |
| HIV diagnoses per 100,000 people aged at least 13 years | 0.00-168.03 | 3.93 (10.62) | **<0.0001** |
| *Covariates* |  |  |  |
| *Healthcare Context* |  |  |  |
| *HIV-Related Healthcare Access* |  |  |  |
| Syringe exchange programs per 100,000 people | 0.00-20.26 | 0.11 (0.75) | **<0.0001** |
| Ryan White HIV medical providers per 100,000 people | 0.00-22.71 | 0.17 (1.09) | **<0.0001** |
| Substance abuse facilities offering all 3 medication assisted treatment services per 100,000 people | 0.00-31.51 | 0.08 (0.71) | **<0.0001** |
| Substance abuse facilities offering HIV testing and accepting Medicaid per 100,000 people | 0.00-59.93 | 0.88 (2.79) | **<0.0001** |
| Percent of population with PrEP indicators receiving a PrEP prescription | 8.10-62.10 | 26.70 (14.34) | 0.14 |
| Medical and diagnostic laboratories per 100,000 people | 1.00-54.00 | 7.15 (5.11) | 0.03 |
| *Social Context* |  |  |  |
| *Demographic Characteristics* |  |  |  |
| Percent of population female | 27.28-57.19 | 49.77 (2.44) | **<0.0001** |
| Percent of population aged 18 to 29 years | 4.33-52.64 | 14.43 (4.10) | **<0.0001** |
| Percent of population non-Hispanic Black | 0.00-87.15 | 7.73 (13.87) | **<0.0001** |
| Percent of population Hispanic | 0.00-99.17 | 8.54 (13.35) | **<0.0001** |
| Percent of population with same-sex unmarried partner | 0.00-0.85 | 0.07 (0.08) | **<0.0001** |
| Percent of population with same-sex spouse | 0.00-1.16 | 0.11 (0.11) | **<0.0001** |
| County population aged at least 13 years | 87.00-820,521.00 | 33,913.61 (52,087.22) | **<0.0001** |
| County population density (number of people per square mile of land area) | 0.04-10,556.80 | 99.20 (366.37) | **<0.0001** |
| Rural-Urban Continuum Code |  |  |  |
| Counties in metro areas of 1 million population or more | 241 (8.63%) |  | **<0.0001** |
| Counties in metro areas of 250,000 to 1 million population | 254 (9.09%) |  |  |
| Counties in metro areas of fewer than 250,000 population | 324 (11.60%) |  |  |
| Urban population of 20,000 or more, adjacent to a metro area | 214 (7.66%) |  |  |
| Urban population of 20,000 or more, not adjacent to a metro area | 91 (3.26%) |  |  |
| Urban population of 2,500 to 19,999, adjacent to a metro area | 593 (21.22%) |  |  |
| Urban population of 2,500 to 19,999, not adjacent to a metro area | 433 (15.50%) |  |  |
| Completely rural or less than 2,500 urban population, adjacent to a metro area | 220 (7.87%) |  |  |
| Completely rural or less than 2,500 urban population, not adjacent to a metro area | 424 (15.18%) |  |  |
| *Social Determinant of Health Factors of Interest* |  |  |  |
| *Economic Context* |  |  |  |
| Percent of population aged at least 16 years unemployed | 0.00-27.15 | 5.28 (2.76) | 0.01 |
| Gini Index^b^ | 0.30-0.71 | 0.44 (0.04) | **<0.0001** |
| Percent of households receiving food stamps/SNAP in past 12 months | 0.00-59.22 | 12.97 (6.54) | **<0.0001** |
| Percent of renter-occupied housing units with rent of at least 30% of household income | 0.00-88.10 | 42.78 (9.78) | **<0.0001** |
| Percent of median income spent on childcare costs for household with two children | 10.15-60.69 | 25.23 (6.28) | 0.06 |
| Total number of child day care services per 100,000 people | 4.00-254.00 | 26.49 (17.43) | 0.74 |
| *Education Context* |  |  |  |
| Percent of population aged at least 25 years with less than high school education | 1.12-73.56 | 13.31 (6.37) | **<0.0001** |
| *Healthcare Context* |  |  |  |
| Percent of population with no health insurance coverage | 0.00-46.30 | 9.75 (5.21) | 0.01 |
| Total number of home healthcare services per 100,000 people | 2.00-166.00 | 14.06 (13.48) | **<0.0001** |
| Presence of a Medically Underserved Area |  |  |  |
| Yes | 1,497 (53.58%) |  | **<0.0001** |
| No | 1,297 (46.42%) |  |  |
| *Physical Infrastructure* |  |  |  |
| Food Environment Index^c^ | 0.00-10.00 | 7.38 (1.16) | **<0.0001** |
| Percent of housing units lacking complete kitchen or plumbing facilities | 0.00-36.81 | 1.27 (1.72) | **<0.0001** |
| Percent of housing units that are overcrowded | 0.00-46.96 | 2.35 (2.39) | **<0.0001** |
| Percent of occupied housing units that are rented | 6.94-100.00 | 27.49 (7.67) | **<0.0001** |
| Percent of housing units with no vehicle available | 0.00-87.99 | 6.12 (4.00) | **<0.0001** |
| Total jail population rate per 100,000 people | 0.00-26,654.55 | 642.80 (1,450.67) | **<0.0001** |
| Index of Dissimilarity^d^ | 0.15-91.63 | 30.01 (13.19) | **<0.0001** |
| *Social Context* |  |  |  |
| Total number of social associations per 100,000 people | 0.00-554.02 | 117.71 (61.04) | **<0.0001** |

^a^Bolded p-values are statistically significant using the Bonferroni corrected significance threshold of α=0.00048; ^b^Gini Index ranges from 0 (best) to 1 (worst); ^c^Food Environment Index ranges from 0 (least healthy) to 10 (most healthy); ^d^Index of Dissimilarity ranges from 0 (best) to 100 (worst)
