# Supplementary material for: Social Determinants of Health and HIV Diagnosis Rates in U.S. Counties, Comparing Ending the Epidemic (EHE) and Non-EHE Priority Jurisdictions
Source: AIDS Behav. Author manuscript; Available in PMC 2025 Oct 10. (PMC12509699; doi:10.1007/s10461-025-04865-x)
Supplement: Supplementary file 4 [file NIHMS2110400-supplement-Supplemental_Table_4.docx]

Table S4. Analytic Sample Description

| Geographic Area | # of Counties: Total Analytic Sample | # of Counties: Non-EHE Priority Jurisdiction Areas | # of Counties: EHE Priority Jurisdiction Areas |
| --- | --- | --- | --- |
| Alabama | 6 | 0 | 6 |
| Arizona | 5 | 4 | 1 |
| Arkansas | 3 | 0 | 3 |
| California | 30 | 22 | 8 |
| Colorado | 7 | 7 | 0 |
| Florida | 31 | 24 | 7 |
| Georgia | 19 | 15 | 4 |
| Illinois | 4 | 3 | 1 |
| Indiana | 10 | 9 | 1 |
| Iowa | 3 | 3 | 0 |
| Kansas | 3 | 3 | 0 |
| Kentucky | 3 | 0 | 3 |
| Louisiana | 8 | 6 | 2 |
| Maryland | 9 | 6 | 3 |
| Massachusetts | 5 | 4 | 1 |
| Michigan | 8 | 7 | 1 |
| Minnesota | 5 | 5 | 0 |
| Mississippi | 5 | 0 | 5 |
| Missouri | 7 | 0 | 7 |
| Nebraska | 2 | 2 | 0 |
| Nevada | 2 | 1 | 1 |
| New Jersey | 16 | 14 | 2 |
| New Mexico | 4 | 4 | 0 |
| New York | 14 | 13 | 1 |
| North Carolina | 18 | 17 | 1 |
| Ohio | 14 | 11 | 3 |
| Oklahoma | 3 | 0 | 3 |
| Oregon | 5 | 5 | 0 |
| Pennsylvania | 17 | 16 | 1 |
| South Carolina | 9 | 0 | 9 |
| Tennessee | 9 | 8 | 1 |
| Texas | 28 | 23 | 5 |
| Utah | 3 | 3 | 0 |
| Virginia | 13 | 13 | 0 |
| Washington | 8 | 7 | 1 |
| Washington D.C. | 1 | 0 | 1 |
| West Virginia | 1 | 1 | 0 |
| Wisconsin | 6 | 6 | 0 |
